# Clinical phenotypes of psychosis and reoffending risk among justice-involved adolescents: a population-based cohort study

**DOI:** 10.3389/fpsyt.2025.1663025

**Published:** 2025-10-22

**Authors:** Emaediong I. Akpanekpo, Tony Butler

**Affiliations:** School of Population Health, The University of New South Wales (UNSW) Sydney, Sydney, NSW, Australia

**Keywords:** adolescents, psychosis, schizophrenia, reoffending, recidivism

## Abstract

**Background:**

High reoffending rates among justice-involved adolescents necessitate identifying modifiable risk factors. Given the elevated prevalence of psychosis in this group compared to the general adolescent population, determining its contribution requires differentiation by clinical phenotypes and quantification of associated risk. This study aimed to determine the association between clinical phenotypes of psychosis and reoffending among justice-involved adolescents.

**Method:**

We conducted a retrospective cohort study using linked administrative health and justice data (2001-2021) from New South Wales, Australia. Justice-involved adolescents (10–17 years) with a diagnosed psychotic disorder identified from hospital/emergency records (n=236) were matched (approx. 1:3) on age, sex, and index offense year to controls without psychosis (n=679). Time to first general and violent reoffending conviction within 24 months was analyzed using stratified Cox proportional hazards models, adjusting for confounders.

**Results:**

Compared to controls, schizophrenia-related psychosis was significantly associated with increased hazards of general reoffending (adjusted Hazard Ratio [aHR]=1.51, 95% CI: 1.10–2.07) and violent reoffending (aHR=2.71, 95% CI: 1.87–3.94). Substance-induced psychosis also showed significantly increased hazards for general reoffending (aHR=3.09, 95% CI: 1.73–5.50) and violent reoffending (aHR=2.87, 95% CI: 1.49–5.56).

**Conclusions:**

Non-affective psychoses significantly elevate reoffending risk among justice-involved adolescents, with different magnitudes of risk associated with schizophrenia-related and substance-induced psychosis. Findings highlight the need for nuanced risk assessment and targeted strategies, including routine screening, early intervention, and integrated care pathways within the youth justice system.

## Introduction

High reoffending rates among justice-involved adolescents, often exceeding 50% (1), carry profound social and economic costs. A key contributor is the high prevalence of mental health disorders. In Australia, 81.3% of justice-involved youth meet criteria for a psychiatric disorder, exceeding rates observed among their community peers (2, 3). Within this context, psychotic disorders are markedly overrepresented. Detained adolescents are up to ten times more likely to experience a psychotic illness than community peers, with Australian prevalence estimates in justice-involved youth ranging from 4.8% to 13.6% (2, 3). These issues converge in adolescence, as peak offending rates (4, 5) coincide with the typical age of onset for psychotic disorders (6). A younger age-at-onset of psychosis is associated with negative clinical outcomes, including increased hospitalizations and poor social functioning (7). The onset of psychosis during this critical developmental period often leads to significant functional impairment, disrupting education, peer relationships, and vocational milestones (8–11). These deficits can exacerbate criminogenic risk factors and hinder rehabilitation.

Psychosis includes distinct clinical phenotypes such as schizophrenia-related disorders, substance-induced psychosis, and affective psychosis (12–15). Untreated, psychosis can impair judgment and impulse control. Perceptual distortions, paranoid delusions, and command hallucinations may lead to antisocial or aggressive responses that would not otherwise occur (16–19). The relationship between psychosis and offending is less clear in studies of broad “psychotic-like symptoms” rather than formal diagnoses. Research on detained youth has shown mixed results, with one study finding no link between such symptoms and recidivism (20), while another suggested some symptoms reduced violent reoffending risk (21). These divergent findings highlight the need to distinguish these experiences from clinically diagnosed psychotic disorders when examining reoffending. In contrast, research on formally diagnosed psychotic disorders more consistently supports an association with reoffending, particularly in adults (14, 15). Evidence for adolescents is less extensive, but recent longitudinal research found that a pre-existing psychosis diagnosis significantly increases incarceration risk compared to other mental disorders (22) and is associated with rapid reincarceration (23).

Important gaps persist. There is need to differentiate risk by clinical phenotypes given their potentially distinct profiles, understand interactions with prevalent comorbidities like disruptive behavioral and substance use disorders, and utilize well-defined comparison groups to isolate the specific contribution of psychosis. To address these gaps, this study used linked health and justice records from New South Wales (NSW), Australia, to examine the association between diagnosed psychosis and reoffending in justice-involved adolescents aged 10–17. We compared adolescents with a diagnosed psychotic disorder (cases) to a cohort from the same justice system without such a diagnosis (controls). Given the existing evidence, we hypothesized that diagnosed psychosis would be associated with an increased hazard of reoffending.

Our specific objectives were: 1) to estimate the individual-level association between diagnosed psychosis (overall and by subtype: schizophrenia-related, substance-induced, affective) and the hazard of general and violent reoffending; 2) to investigate potential interactions between psychosis sub-types and key comorbidities (disruptive behavioral disorder and substance use disorder) on reoffending risk; and 3) to identify other factors associated with general and violent reoffending within this cohort to better contextualize the role of psychosis.

## Methods

### Study setting, design, and data sources

This retrospective cohort study utilized routinely collected, linked administrative data from NSW, Australia, comparing justice-involved adolescents with diagnosed psychosis to a matched control group drawn from the same justice system context. Probabilistic record linkage was performed by the NSW Centre for Health Record Linkage (CHeReL) using validated protocols (estimated false positive linkage rate <0.5%). Linked datasets included: the Admitted Patient Data Collection (APDC, 2001–2021), providing information on all hospital admissions including diagnoses coded using the International Classification of Diseases, Tenth Revision, Australian Modification (ICD-10-AM); the Emergency Department Data Collection (EDDC, 2005–2021), documenting emergency presentations, including diagnoses recorded using Systematized Nomenclature of Medicine - Clinical Terms (SNOMED-CT) which were mapped to ICD classifications; the NSW Bureau of Crime Statistics and Research’s Re-offending Database (ROD, 2001–2020), providing information on court appearances, charges, convictions, sentencing, and individual demographics; and the NSW Registry of Births, Deaths, and Marriages (RBDM, 2001–2021), for mortality ascertainment.

### Study population and cohort construction

The study cohort was drawn from adolescents involved with the NSW youth justice system. Eligibility required being aged 10 to 17 years at the time of a proven index offense (i.e., one resulting in a conviction), as recorded in the NSW Bureau of Crime Statistics and Research’s Reoffending Database (ROD), in alignment with the legal criteria for youth justice involvement.

Cases were individuals within the source population who had a recorded diagnosis of a psychotic disorder, identified from linked APDC or EDDC records, occurring on or before the date of their index offense. Psychosis diagnoses were classified based on ICD-10-AM codes. Schizophrenia and related psychosis were identified using ICD-10 codes: F06.0, F06.2, F20.0-F20.9, F22.0, F22.8, F22.9, F23.0-F23.3, F23.8, F23.9, F24, F25.0-F25.2, F25.8, F25.9, F28, F29, F53. Affective psychosis included ICD-10 codes: F30.2, F31.2, F31.5, F32.3, F33.3. Substance-induced psychosis included ICD-10 codes: F10.5, F11.5, F12.5, F13.5, F14.5, F15.5, F16.5, F17.5, F18.5, F19.5. Consistent with prior research (14), a hierarchical framework ensured mutually exclusive categorization: 1) Schizophrenia and related psychosis; 2) Affective psychosis in the absence of schizophrenia and related psychosis; 3) Substance-induced psychosis in the absence of the other two clinical variants.

Controls were selected from a pool of young people under Youth Justice NSW supervision (in custody or community orders) who participated in health surveys conducted between 2003 and 2015: the Young People in Custody Health Survey (YPiCHS; conducted in 2003, 2009, and 2015) and the Young People on Community Orders Health Survey (YPoCoHS; conducted 2003–2006) (24–27). These surveys employed total population sampling methodology. Response rates exceeded 90% for youth in custody, and previous studies utilizing these surveys demonstrated that post-stratification weighting did not materially change estimates, supporting the representativeness of the sample (28, 29).

Like cases, these potential controls were aged 10–17 years at the time of their index offense occurring around the time of survey enrolment. Individual survey data were linked by CHeReL to the same administrative datasets (ROD, APDC, EDDC, RBDM) used for cases. Individuals were eligible as controls if they had no recorded diagnosis of a psychotic disorder in the APDC or EDDC data on or before the date of the index offense of their matched case. Matching criteria were age (± 6 months), sex and calendar year of index offense (± 1 year). The final matched cohort included 236 cases and 679 controls (match ratio approximately 1:3).

### Ethics approval

This study was conducted according to the guidelines of the Declaration of Helsinki. All procedures involving human participants were approved by the NSW Population & Health Services Research Ethics Committee (Refs: 2019/ETH01721; 2019/ETH13028), the Aboriginal Health & Medical Research Council Ethics Committee (Refs: 1089/15; 1394/18), the Justice Health and Forensic Mental Health Network Human Research Ethics Committee (Refs: G324/14; G692/15), and the Corrective Services NSW Ethics Committee (Ref: D2023/1555831). Informed consent to participate was obtained from all individuals whose data was collected via surveys. For the current study, which involved the secondary analysis and linkage of this survey data with de-identified administrative records, a waiver of the requirement to seek further project-specific consent was granted by the NSW Population & Health Services Research Ethics Committee. This waiver was granted as the research involved no active participation or re-contact with participants.

### Exposures

The primary exposure was the presence of a diagnosed psychotic disorder, ascertained at the index offense date based on diagnoses recorded in linked hospital admission (APDC) or emergency department (EDDC) data. A secondary exposure variable, psychosis subtype, categorized cases according to the hierarchical classification: schizophrenia-related, affective, or substance-induced psychosis.

### Outcomes

The primary outcome was the time from the index offense to any subsequent convicted re-offense within a 24-month follow-up period. The secondary outcome was the time to a violent re-offense within a 24-month follow-up period. Convicted offenses were used instead of charges or arrests to provide a more reliable measure of reoffending as it excludes cases that were dismissed or resulted in acquittal. Follow-up began on the date of index offense conviction and ended at the earliest of: first re-offense conviction, death, or 24 months post-index offense. A 24-month follow-up period was chosen to allow sufficient opportunity to observe reoffending events, consistent with studies on recidivism (30). Reoffending data were obtained from the ROD.

### Measures and definitions

Violent offenses were defined according to categories 01 to 06 of the Australian and New Zealand Standard Offence Classification (ANZSOC) (31). This includes offenses involving physical harm, threats of violence, or coercive actions against others: homicide and related offenses (01), acts intended to cause injury (02), sexual assault and related offenses (03), dangerous or negligent acts endangering persons (04), abduction, harassment, and other offenses against the person (05), and robbery, extortion, and related offenses (06). ‘Previous remand history’ was ascertained from the ROD, indicating any recorded police- or court-ordered placement in custody on remand prior to the index offense date.

‘Indigenous status’ was defined as identification as an Aboriginal and/or Torres Strait Islander person, based on records within administrative health or justice datasets. ‘Socio-economic disadvantage’ was derived from the Australian Bureau of Statistics’ Index of Relative Socio-economic Disadvantage (IRSD) score for the individual’s residential area postcode at index offense, grouped into population-based quartiles (Q1 = most disadvantaged, Q4 = least disadvantaged).

‘Disruptive behavioral disorder’ was defined as a recorded diagnosis (from APDC or EDDC) related to Hyperkinetic disorders (F90), Conduct disorders (F91), and Mixed disorders of conduct and emotions (F92), identified based on any listed diagnostic code within these categories during admission to hospital or emergency department presentation. ‘Trauma/stress-related disorder’ was defined as a recorded diagnosis (from APDC or EDDC) under F43 (Reaction to severe stress, and adjustment disorders), identified based on any listed diagnostic code within these categories during admission to hospital or emergency department presentation. ‘Substance use disorder (excluding substance-induced psychosis)’ was defined as a recorded diagnosis (from APDC or EDDC) within the ICD-10 F10–F19 block (Mental and behavioral disorders due to psychoactive substance use), excluding codes which denote substance-induced psychotic disorders. This was identified based on admission to hospital or emergency department presentation. Substance-induced psychosis captures the acute psychotic state directly triggered by substance use, while the broader substance use disorder variable (excluding psychosis codes) captures patterns of problematic substance use independent of psychotic symptoms.

### Statistical analysis

Baseline characteristics of the matched cohort were summarized, with frequencies and percentages reported for categorical variables and medians (interquartile ranges, IQR) for continuous variables. Baseline was the date of the index offense. Differences between the psychosis and non-psychosis groups at baseline were assessed using chi-squared tests for categorical variables, and Mann-Whitney U tests for continuous variables. Time-to-event analyses utilized Kaplan-Meier methods to generate survival curves for time to first court-proven reoffending conviction over the 24-month follow-up period. Survival distributions were compared between groups using the log-rank test.

Stratified Cox proportional hazards regression was employed to estimate the association between psychosis and time to reoffending. Analyses were stratified by the unique matched-set identifier, which non-parametrically controlled for the matching. Covariates included in the final adjusted models were selected *a priori* based on review of relevant literature and their theoretical potential to confound the association between psychosis and reoffending (14, 22, 23). These included: Indigenous status (binary), socio-economic disadvantage (IRSD quartiles, categorical), concurrent offenses (continuous), violent index offense status (binary), previous remand history (binary), disruptive behavioral disorder (binary), trauma/stress-related disorder (binary), and substance use disorder (non-psychotic, binary).

Because analyses were stratified by matched sets to control for matching factors (age ± 6 months, sex, index offense year ± 1 year), these variables were not included as covariates in the models to avoid collinearity with the stratification structure. Separate models were run using either overall psychosis status or psychosis subtype indicators as the exposure variable(s), relative to the control group. Adjusted hazard ratios (aHRs) and 95% confidence intervals (CIs) were reported as measures of association. The proportional hazards assumption for the final models was assessed using Schoenfeld residuals and was found to hold (p>0.05 based on the global test). As exploratory analyses, we tested for interactions between psychosis subtypes and diagnoses of disruptive behavioral disorder and substance use disorder by including multiplicative interaction terms in the main multivariable Cox regression models. Further exploratory analyses tested for interactions between overall psychosis status (case *vs*. control) and having a violent index offense (binary) for both general and violent reoffending outcomes.

The mechanism of missing data was investigated using Little’s test, which failed to reject the null hypothesis that data were Missing Completely at Random (MCAR; p>0.05) (32). As only a negligible proportion of observations (n = 19, 2.1%) had missing data for variables included in the final models, complete case analysis was performed. This approach is considered unlikely to introduce substantial bias under the MCAR assumption (33). All statistical analyses were performed using Stata version 19.5 (StataCorp LLC, College Station, TX, USA). A two-sided p-value < 0.05 was considered statistically significant.

## Results

### Cohort characteristics

The final matched cohort included 915 justice-involved adolescents (236 with psychosis [cases] and 679 without psychosis [controls], matched approximately 1:3). Baseline characteristics of the cohort are presented in **Table 1**. The median age at index offense was 16 years (IQR: 15–17) and the mean age was 15.7 years (SD = 1.2) for both cases and controls, with no significant difference in age distribution between the groups (p=0.854). There was no significant difference in the sex distribution between groups (p=0.763), with males comprising 82.2% of cases and 83.1% of controls. Within the psychosis group (n=236), schizophrenia-related disorders were the most common subtype (n=183, 77.5%), followed by substance-induced psychosis (n=45, 19.1%) and affective psychosis (n=8, 3.4%).

**Table 1:** Characteristics of the study population (n = 915).

Characteristics	Total (N = 915)	Psychosis cases (n=236)	Controls (n=679)	P value
Age (years)				0.854
Median (IQR)	16 (15, 17)	16 (15, 17)	16 (15, 17)	
Mean (SD)	15.7 ± 1.2	15.7 ± 1.2	15.7 ± 1.2	
Sex				0.763
Female	157 (17.2)	42 (17.8)	115 (16.9)	
Male	758 (82.8)	194 (82.2)	564 (83.1)	
Indigenous status				0.030
Indigenous	433 (47.3)	126 (53.4)	307 (45.2)	
Non-Indigenous	482 (52.7)	110 (46.6)	372 (54.8)	
IRSD Quartiles				0.042
Q1	318 (34.8)	65 (27.5)	253 (37.3)	
Q2	229 (25.0)	70 (29.7)	159 (23.4)	
Q3	253 (27.7)	62 (26.3)	191 (28.1)	
Q4	96 (10.5)	28 (11.9)	68 (10.0)	
Missing	19 (2.1)			
Index offense				<0.001
Non-violent	544 (59.5)	165 (69.9)	379 (55.8)	
Violent	371 (40.6)	71 (30.1)	300 (44.2)	
Concurrent offenses				0.092
Median (IQR)	2 (1, 4)	2 (1, 4)	2 (1, 3)	
Mean (SD)	3.3 ± 3.9	3.6 ± 3.8	3.1 ± 3.9	
Previous remand				
≥1 remand episode	379 (41.4)	42 (17.8)	337 (49.6)	<0.001
Mental health disorders				
Disruptive behavioral disorder	121 (13.2)	94 (39.8)	27 (4.0)	<0.001
Substance use disorder (excluding substance-induced psychosis)	203 (22.2)	102 (43.2)	101 (14.9)	<0.001
Trauma/stress-related disorder	85 (9.3)	48 (20.3)	37 (5.4)	<0.001
Psychosis subtypes (cases only) ^†^				
Schizophrenia-related psychosis	–	183 (77.5)	–	–
Affective psychosis	–	8 (3.4)	–	–
Substance-induced psychosis	–	45 (19.1)	–	–
Follow-up period				
Follow-up time, Median (IQR)	14 (4, 24)	6 (2, 20)	18 (6, 24)	
General reoffending	557 (60.9)	179 (75.8)	378 (55.7)	<0.001
Violent reoffending	339 (37.0)	135 (57.2)	204 (30.0)	<0.001

†Psychosis subtype percentages calculated based on psychosis cases column total (n=236). IQR, Interquartile range; SD, Standard deviation.

### Time to general reoffending

**Figure 1:**
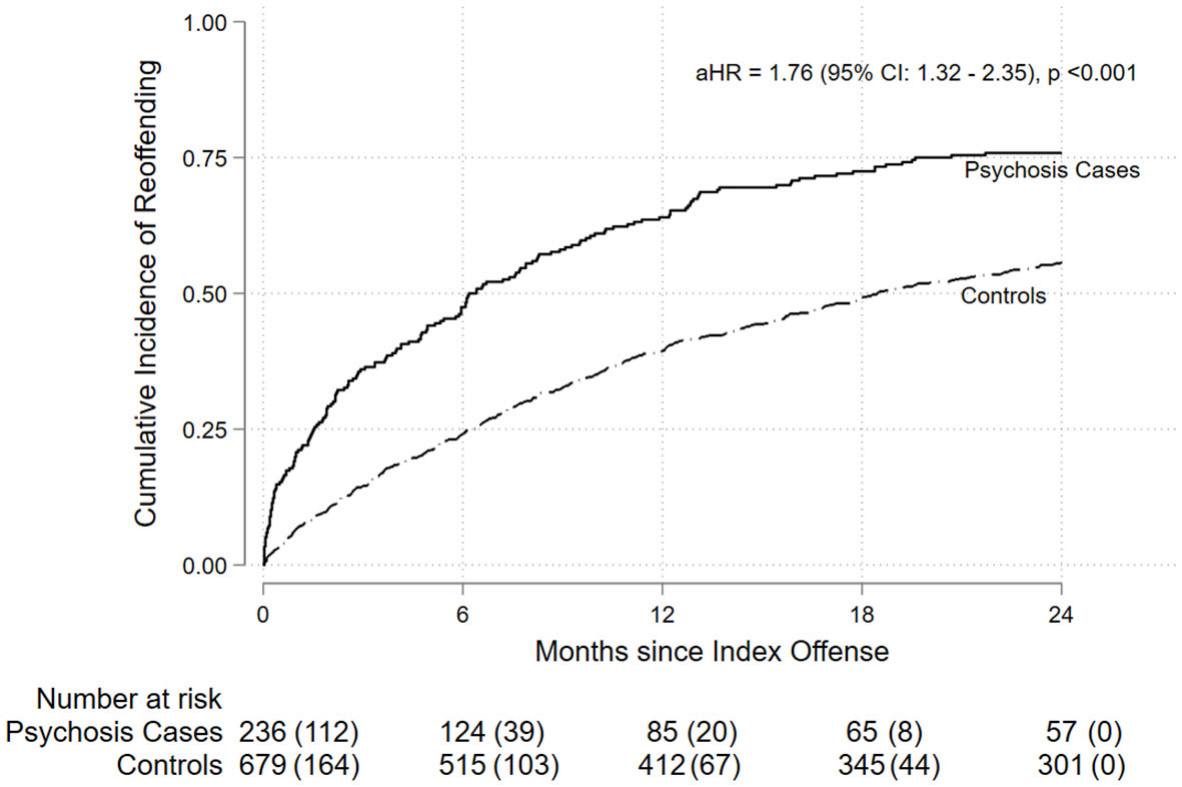
Cumulative incidence of general reoffending over 24 months from the index offense (x-axis: Months since index offense; y-axis: Cumulative incidence of reoffending). Adolescents with psychosis showed a higher cumulative incidence than matched controls (log-rank p < 0.001). Numbers below the plot show individuals at risk, with cumulative events in parentheses.

During the 24-month follow-up period, 60.9% of the full matched cohort experienced a reoffending conviction. Based on the principal (first recorded) general re-offense, the most common types across the full cohort were acts intended to cause injury (14.9%), theft and related offenses (9.5%), and offenses against government procedures, security and operations (8.9%) (**Supplementary Table S1**). The incidence proportion of general reoffending was higher among cases with psychosis (75.8%) compared to controls without psychosis (55.7%). Examining the incidence proportion by psychosis subtype revealed the highest incidence for substance-induced psychosis (80.0%) and schizophrenia-related psychosis (75.4%), followed by affective psychosis (62.5%). Kaplan-Meier analysis indicated a significantly shorter time to reoffending for adolescents with psychosis compared to controls (log-rank p < 0.001; **Figure 1**).

Consistent with the Kaplan-Meier analysis (**Figure 1**), the adjusted hazard ratio comparing psychosis cases to controls indicated an increased risk (aHR = 1.76, 95% CI: 1.32–2.35). Compared to controls without psychosis, both schizophrenia-related psychosis (aHR = 1.51, 95% CI: 1.10–2.07) and substance-induced psychosis (aHR = 3.09, 95% CI: 1.73–5.50) were associated with an increased hazard of reoffending (**Table 2**).

**Table 2:** Univariable and multivariable Cox regression analyses for general reoffending.

Characteristics	Univariable analysis		Multivariable analysis	
	aHR (95% CI)	P value	aHR (95% CI)	P value
Psychosis type				
Control group	1.0 (ref)		1.0 (ref)	
Schizophrenia-related disorder	1.82 (1.44–2.30)	<0.001	1.51 (1.10–2.07)	0.011
Substance-induced psychosis	3.72 (2.20–6.28)	<0.001	3.09 (1.73–5.50)	<0.001
Affective psychosis	4.32 (1.03–18.18)	0.046	2.78 (0.63–12.30)	0.178
Indigenous status				
Non-Indigenous	1.0 (ref)		1.0 (ref)	
Indigenous	1.56 (1.25–1.95)	<0.001	1.42 (1.12–1.80)	0.004
Socio–economic disadvantage				
Q1 (Most disadvantaged)	1.0 (ref)		1.0 (ref)	
Q2	1.21 (0.91–1.60)	0.183	1.02 (0.76–1.38)	0.875
Q3	1.10 (0.84–1.44)	0.510	1.03 (0.77–1.37)	0.855
Q4 (Least disadvantaged)	1.14 (0.78–1.67)	0.505	1.10 (0.74–1.66)	0.633
Concurrent offenses				
Per unit increase	1.05 (1.02–1.08)	<0.001	1.06 (1.02–1.09)	<0.001
Index offense				
Non-violent	1.0 (ref)		1.0 (ref)	
Violent	0.66 (0.53–0.82)	<0.001	0.70 (0.55–0.89)	0.003
Previous remand				
No previous remand	1.0 (ref)		1.0 (ref)	
Previous remand	0.83 (0.66–1.03)	0.091	0.89 (0.68–1.16)	0.378
Disruptive behavioral disorder				
Not Present	1.0 (ref)		1.0 (ref)	
Present	2.42 (1.79–3.29)	<0.001	1.48 (1.01–2.17)	0.044
Trauma/stress-related disorder				
Not Present	1.0 (ref)		1.0 (ref)	
Present	1.54 (1.07–2.21)	0.020	1.06 (0.69–1.62)	0.793
Substance use disorder				
Not Present	1.0 (ref)		1.0 (ref)	
Present	1.58 (1.22–2.04)	<0.001	1.09 (0.80–1.48)	0.580

aHR, adjusted Hazard Ratio; CI, Confidence Interval; All analyses were conducted using stratified Cox regression models (stratified by match set ID).

Other factors significantly associated with an increased hazard of general reoffending in the multivariable model included Indigenous status (aHR = 1.42, 95% CI: 1.12–1.80), a higher number of concurrent offenses (aHR per unit in-crease = 1.06, 95% CI: 1.02–1.09), and presence of a disruptive behavioral disorder (aHR = 1.48, 95% CI: 1.01–2.17). Having a violent index offense (*vs*. non-violent) was associated with a lower hazard of general reoffending (aHR = 0.70, 95% CI: 0.55–0.89).

### Time to violent reoffending

**Figure 2:**
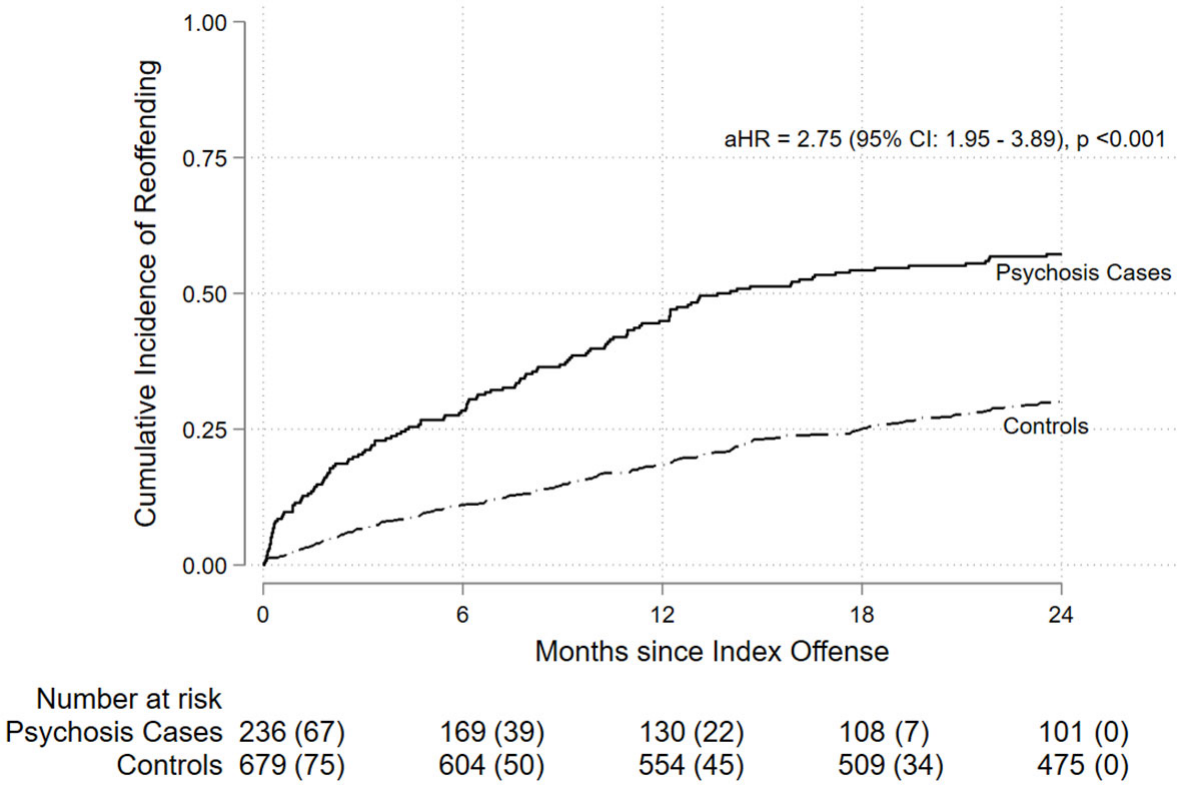
Cumulative incidence of violent reoffending over 24 months from the index offense (x-axis: Months since index offense; y-axis: Cumulative incidence of violent reoffending). Adolescents with psychosis showed a higher cumulative incidence than matched controls (log-rank p < 0.001). Numbers below the plot show individuals at risk, with cumulative events in parentheses.

A total of 37.0% of the full matched cohort experienced a violent reoffending conviction during the 24-month follow-up. Based on the principal (first recorded) violent re-offense, the most common types across the full cohort were acts intended to cause injury (25.4%) and robbery, extortion and related offenses (7.3%) (**Supplementary Table S2**). Notably, acts intended to cause injury accounted for 44.5% of violent offenses among cases compared to 18.7% among controls. The incidence proportion of violent reoffending was higher for cases with psychosis (57.2%) than for controls (30.0%). By subtype, the incidence proportions were 59.0% for schizophrenia-related psychosis, 51.1% for substance-induced psychosis, and 50% for affective psychosis. Kaplan-Meier analysis indicated that adolescents with psychosis had a significantly shorter time to violent reoffending compared to controls (log-rank p < 0.001; **Figure 2**).

Consistent with the Kaplan-Meier analysis (**Figure 2**), the adjusted hazard ratio comparing psychosis cases to controls indicated a significantly increased risk of violent reoffending (aHR = 2.75, 95% CI: 1.95–3.89). In the multivariable stratified Cox model (**Table 3**), both schizophrenia-related psychosis (aHR = 2.71, 95% CI: 1.87–3.94) and substance-induced psychosis (aHR = 2.87, 95% CI: 1.49–5.56) were associated with an increased hazard of violent reoffending compared to controls without psychosis.

**Table 3:** Univariable and multivariable Cox regression analyses for violent reoffending.

Characteristics	Univariable analysis		Multivariable analysis	
	aHR (95% CI)	P value	aHR (95% CI)	P value
Psychosis type				
Control group	1.0 (ref)		1.0 (ref)	
Schizophrenia-related	2.54 (1.93–3.34)	<0.001	2.71 (1.87–3.94)	<0.001
Substance-induced psychosis	3.14 (1.72–5.74)	<0.001	2.87 (1.49–5.56)	0.002
Affective psychosis	3.37 (0.75–15.18)	0.113	3.35 (0.70–16.14)	0.131
Indigenous status				
Non-Indigenous	1.0 (ref)		1.0 (ref)	
Indigenous	1.61 (1.23–2.11)	0.001	1.45 (1.08–1.95)	0.014
Socio–economic disadvantage				
Q1 (Most disadvantaged)	1.0 (ref)		1.0 (ref)	
Q2	1.07 (0.77–1.51)	0.682	0.80 (0.56–1.16)	0.245
Q3	1.18 (0.84–1.64)	0.337	1.11 (0.78–1.58)	0.554
Q4 (Least disadvantaged)	1.38 (0.86–2.19)	0.179	1.41 (0.86–2.32)	0.170
Concurrent offenses				
Per unit increase	1.07 (1.03–1.11)	<0.001	1.07 (1.02–1.11)	0.002
Index offense				
Non-violent	1.0 (ref)		1.0 (ref)	
Violent	0.85 (0.65–1.11)	0.238	0.98 (0.73–1.31)	0.875
Previous remand				
No previous remand	1.0 (ref)		1.0 (ref)	
Previous remand	0.70 (0.53–0.91)	0.009	0.91 (0.66–1.25)	0.573
Disruptive behavioral disorder				
Not Present	1.0 (ref)		1.0 (ref)	
Present	2.30 (1.63–3.26)	<0.001	1.16 (0.74–1.81)	0.521
Trauma/stress-related disorder				
Not Present	1.0 (ref)		1.0 (ref)	
Present	1.32 (0.86–2.03)	0.205	0.86 (0.51–1.44)	0.558
Substance use disorder				
Not Present	1.0 (ref)		1.0 (ref)	
Present	1.28 (0.95–1.74)	0.111	0.77 (0.53–1.12)	0.170

aHR, adjusted Hazard Ratio; CI, Confidence Interval; All analyses were conducted using stratified Cox regression models (stratified by match set ID).

Other factors significantly associated with an increased hazard of violent reoffending in the multivariable model (**Table 3**) were Indigenous status (aHR = 1.45, 95% CI: 1.08–1.95) and a higher number of concurrent offenses (aHR per unit increase = 1.07, 95% CI: 1.02–1.11).

### Exploratory analyses

Exploratory analyses revealed two significant interactions. First, there was a significant interaction between disruptive behavioral disorders and substance-induced psychosis for violent reoffending (p=0.036). In this model, adolescents with either diagnosis alone had significantly increased risk (disruptive behavioral disorders only: aHR 2.15 [95% CI: 1.38–3.36]; substance-induced psychosis only: aHR 2.22 [95% CI: 1.22–4.03]), while those with both conditions had a modest, non-significant increase compared to controls (aHR = 1.62 [95% CI: 0.77–3.44]) (**Supplementary Figure S1**).

Second, further analyses revealed a significant interaction between overall psychosis status and index offense type (violent *vs*. non-violent) for both general reoffending (p = 0.022) and violent reoffending (p = 0.037). The joint effects are displayed in **Supplementary Figure S2**. For general reoffending, compared to controls with a non-violent index offense, risk was significantly increased only for the psychosis/non-violent index group (aHR=2.21 [1.56–3.14]); risk for the psychosis/violent index group was not significantly different (aHR=0.93 [0.59–1.47]). For violent reoffending, compared to the same reference group, risk was significantly increased for both the psychosis/non-violent index group (aHR=3.56 [2.33–5.43]) and the psychosis/violent index group (aHR=2.11 [1.23–3.61]).

## Discussion

This study examined the association between diagnosed psychotic disorders and reoffending among justice-involved adolescents, utilizing linked administrative data and a matched cohort design. Adolescents with a psychotic disorder diagnosis faced a significantly higher risk of both general and violent reoffending over a 24-month period compared to matched controls without psychosis, even after adjusting for key confounders. Further analyses revealed heterogeneity across clinical phenotypes. Schizophrenia-related psychosis was associated with increased hazards for both general and violent reoffending, while substance-induced psychosis demonstrated the highest risk for general reoffending and a comparably elevated risk for violent reoffending.

Situating our findings requires consideration of the broader literature, particularly given the limited research examining psychosis and reoffending in adolescent populations. Some studies focusing on psychotic-like symptoms in detained youth have yielded mixed findings. For example, a previous study reported no significant association between the presence of psychotic-like symptoms and either general or violent recidivism among detained boys (20). Other studies have found that psychotic-like symptoms were associated with reduced risk of violent recidivism (21). Differences between our findings based on formal diagnoses and prior studies focusing on self-reported psychotic-like symptoms might be attributable to differences in measurement (diagnosis *vs*. symptoms) and potentially reflect that administratively recorded diagnoses capture clinically recognized presentations of psychosis compared to broader symptom screeners. More pertinent are longitudinal studies of diagnosed psychosis. Notably, a previous longitudinal study found that psychosis in justice-involved adolescents increased the risk of subsequent incarceration compared to adolescents with other mental disorders (22). Similarly, another study demonstrated that psychosis in justice-involved adolescents was associated with reincarceration (23).

Our results broadly resonate with adult literature, where psychosis has been shown to increase reoffending risk (14, 34), particularly when comorbid with substance use (15). Consistent with this, a recent study found no differences in neurological soft signs between adults with schizophrenia who had a history of violence and those without, but reported higher rates of substance misuse, poorer medication adherence, lower educational attainment, and more intensive antipsychotic treatment in the violent group (35). These adult findings suggest that, in the context of schizophrenia, factors such as treatment engagement and illness trajectory may be more informative when evaluating or interpreting risk. This interpretation is further supported by evidence from justice-involved adolescents with psychosis, where the absence of early mental health treatment was associated with higher violent reoffending risk, and where reoffending risk followed a similar gradient by clinical subtype—highest in substance-induced psychoses (36). Taken together, findings from both adolescent and adult populations point to a convergence of evidence linking treatment engagement and clinical phenotype with reoffending risk in the context of psychosis.

In the multivariable analyses, the effect size of disruptive behavioral disorder warrants specific discussion due to complex findings. Consistent with its established role as a risk factor for delinquency (37–40), a diagnosis of disruptive behavioral disorder was associated with an increased risk of general reoffending. Regarding violent reoffending, a significant interaction emerged between disruptive behavioral disorders and substance-induced psychosis. Adolescents with disruptive behavioral disorders only had an increased risk of violent reoffending compared to those with neither disruptive behavioral disorder nor substance-induced psychosis. Similarly, adolescents with substance-induced psychosis only had an increased risk of violent reoffending compared to those with neither substance-induced psychosis nor disruptive behavioral disorder. This indicates that, on average, both diagnoses independently contribute to an increased hazard of violent reoffending. However, the significant interaction between these two diagnoses (i.e., disruptive behavioral disorder and substance-induced psychosis) demonstrates that their combined effect is significantly less than multiplicative. The sub-multiplicative effect suggests the mechanisms driving violence may differ or overlap when both conditions co-occur, attenuating the combined risk.

Index offense type significantly modified the effect of psychosis on reoffending risk. The nature of this modification differed by outcome. For violent reoffending, psychosis was associated with an elevated risk regardless of index offense type, when compared to controls without psychosis and non-violent index offenses. Notably, the magnitude of this increased risk for violent reoffending appeared higher for adolescents with psychosis whose index offense was non-violent compared to those whose index offense was violent, although both groups were at significantly elevated risk relative to controls. In contrast, for general reoffending, the risk effect of psychosis was evident only among individuals with a non-violent index offense. The high risk observed among the psychosis group with non-violent index offenses suggests underlying differences. One plausible interpretation involves differential system responses. Young people presenting with psychosis and non-violent offenses might receive less intensive clinical or supervisory interventions compared to those presenting with overt violence, although this interpretation remains speculative. Alternatively, this subgroup (psychosis with non-violent index offense) may possess distinct clinical profiles or a higher burden of unmeasured criminogenic factors contributing to persistent offending. Understanding the characteristics distinguishing this group warrants further investigation.

In the present study, while the diagnosis of substance-induced psychosis emerged as a risk factor, the presence of a diagnosis of substance use disorder (defined operationally to exclude substance-induced psychosis) was not significantly associated with either reoffending outcome after adjusting for psychosis and other covariates. Furthermore, no significant interactions were identified involving the substance use disorder diagnosis variable. This suggests that, within the analytical framework of the present study, the risk elevation associated with substance use was captured by the acute ‘substance-induced psychosis’ diagnosis, potentially overshadowing the independent contribution of a broader substance use disorder diagnosis when controlling for factors like disruptive behavioral disorder and the psychosis state itself. It does not imply that non-psychotic substance use is irrelevant, but rather that its unique contribution was not statistically significant beyond the effect of substance-induced psychosis and other included variables in the multivariable model.

Beyond psychosis, other factors emerged as risk factors of reoffending. Indigenous status was associated with an increased hazard for both general and violent reoffending. This finding aligns with extensive literature highlighting the disproportionate involvement of Indigenous youth in the justice system, likely reflecting complex interactions between intergenerational trauma, systemic disadvantage, socio-economic factors (though adjusted for in the present study), discrimination, and potentially differing policing or sentencing practices (41–44). A higher number of concurrent offenses at the index event was also associated with both general and violent reoffending. This could indicate that adolescents presenting with severe initial offending patterns are generally more likely to continue offending, possibly reflecting underlying criminogenic needs or a deeper entrenchment in offending behavior not fully captured by other variables.

Furthermore, while factors like previous remand history and substance use disorder (excluding substance-induced psychosis) are often considered established risk factors for recidivism, they were not significantly associated with either general or violent reoffending. Two non-exclusive explanations are plausible: (i) differential system responses for adolescents with psychosis (e.g., diversion, enhanced supervision, or therapeutic dispositions) that attenuate the association between remand history and subsequent reoffending; and (ii) residual confounding and collinearity with proximal markers of offending severity, which may dilute the independent contribution of prior remand noted in earlier work. These possibilities warrant confirmation in future studies with richer measures of system contact and supervision intensity.

### Limitations

Several limitations warrant consideration. Reliance on administrative data introduces constraints. The classification of psychosis based on recorded ICD-10 diagnoses from hospital or emergency department records, while objective, may lead to potential misclassification like undiagnosed cases among controls, potentially biasing observed associations towards the null. Relatedly, hospital and emergency department records likely captured more severe psychosis presentations, potentially missing individuals managed exclusively in community mental health settings. This may bias the sample toward higher clinical severity, limiting generalizability to all justice-involved adolescents with psychosis. If severity independently influences reoffending beyond measured covariates, effect estimates could be biased, though the direction of this bias is uncertain. Generalizability is further constrained to the New South Wales youth justice context. Furthermore, the reoffending outcome was limited to officially documented court reconvictions, likely underestimating the true extent of reoffending behavior and excluding offenses committed outside the state.

Methodological challenges related to selection bias and residual confounding also persist. Although using controls improved baseline balance for age and sex, significant differences persisted, and sourcing controls from survey participants introduces potential selection biases as they may differ systematically from non-participants or the case population. Although the source data for controls were determined to be representative based on response rates and evidence from related studies (24–26, 28, 29), potential differences between survey participants and non-participants in factors associated with reoffending cannot be ruled out.

The multivariable models accounted for measured confounders, however, residual confounding from unmeasured factors is a concern. Additionally, the stratified Cox analysis design precluded separate estimation of the effects of the matching factors (age, sex, index year) due to collinearity. Conclusions regarding affective psychosis cannot be drawn due to the very small subgroup (n=8), which produced imprecise estimates; accordingly, inference in this study is limited to non-affective psychoses (schizophrenia-related and substance-induced). Finally, the study was not formally preregistered. Therefore, while our primary analyses were designed to test *a priori* hypotheses, findings from the interaction analyses should be considered exploratory.

## Conclusion

This study provides evidence that diagnosed non-affective psychosis (schizophrenia-related and substance-induced) is associated with an increased risk of both general and violent reoffending among adolescents involved in the justice system. The findings highlight the importance of recognizing diagnostic heterogeneity and the role of comorbidities, particularly the distinct influence of disruptive behavioral disorders on reoffending, as well as their interaction with substance-induced psychosis. It is evident that addressing the complex clinical needs of justice-involved adolescents with psychosis is essential, necessitating targeted screening, comprehensive assessment, and integrated, culturally responsive interventions. Such efforts are vital for improving outcomes and interrupting pathways to persistent reoffending. Future research should further explore these mechanisms and evaluate the effectiveness of tailored interventions within youth justice settings.

## Data Availability

The data analyzed in this study is subject to the following licenses/restrictions: Due to the sensitive nature of the data and the ethics approvals for this study, the linked de-identified data cannot be shared. Researchers may apply for access to the source datasets through the respective data custodians. Requests to access these datasets should be directed to https://www.cherel.org.au/contact.
