# Supplementary material for: Clinical phenotypes of psychosis and reoffending risk among justice-involved adolescents: a population-based cohort study
Source: Front Psychiatry. 2025 Oct 22;16:1663025. doi: 10.3389/fpsyt.2025.1663025 (PMC12586121; doi:10.3389/fpsyt.2025.1663025)
Supplement: Supplementary file 1 [file Supplementaryfile1.docx]

**Supplementary Table 1. Principal (first recorded) general offense by ANZSOC division, stratified by case status**

| ANZSOC Division | All (n = 915) | Cases (n = 236) | Controls (n = 679) |
| --- | --- | --- | --- |
| Total general offenses | 557 (60.9%) | 179 (75.8%) | 378 (55.7%) |
| Acts intended to cause injury | 136 (14.9%) | 42 (17.8%) | 94 (13.8%) |
| Theft and related offences | 87 (9.5%) | 27 (11.4%) | 60 (8.8%) |
| Offences against government procedures, security and operations | 81 (8.9%) | 39 (16.5%) | 42 (6.2%) |
| Unlawful entry with intent / burglary, break and enter | 56 (6.1%) | 8 (3.4%) | 48 (7.1%) |
| Robbery, extortion and related offences | 45 (4.9%) | 7 (3.0%) | 38 (5.6%) |
| Public order offences | 36 (3.9%) | 20 (8.5%) | 16 (2.4%) |
| Traffic and vehicle regulatory offences | 32 (3.5%) | 1 (0.4%) | 31 (4.6%) |
| Property damage and environmental pollution | 25 (2.7%) | 17 (7.2%) | 8 (1.2%) |
| Dangerous or negligent acts endangering persons | 11 (1.2%) | 2 (0.8%) | 9 (1.3%) |
| Fraud, deception and related offences | 11 (1.2%) | 4 (1.7%) | 7 (1.0%) |
| Illicit drug offences | 11 (1.2%) | 2 (0.8%) | 9 (1.3%) |
| Prohibited and regulated weapons offences | 11 (1.2%) | 4 (1.7%) | 7 (1.0%) |
| Abduction, harassment and other offences against the person | 6 (0.7%) | 3 (1.3%) | 3 (0.4%) |
| Homicide and related offences | 4 (0.4%) | 0 (0.0%) | 4 (0.6%) |
| Miscellaneous offences | 3 (0.3%) | 2 (0.8%) | 1 (0.1%) |
| Sexual assault and related offences | 2 (0.2%) | 1 (0.4%) | 1 (0.1%) |

**Supplementary Table 2. Principal (first recorded) violent offense by ANZSOC division, stratified by case status**

| ANZSOC Division | All (n = 915) | Cases (n = 236) | Controls (n = 679) |
| --- | --- | --- | --- |
| Total violent offenses | 339 (37.0%) | 135 (57.2%) | 204 (30.0%) |
| Acts intended to cause injury | 232 (25.4%) | 105 (44.5%) | 127 (18.7%) |
| Robbery, extortion and related offences | 67 (7.3%) | 16 (6.8%) | 51 (7.5%) |
| Dangerous or negligent acts endangering persons | 22 (2.4%) | 8 (3.4%) | 14 (2.1%) |
| Abduction, harassment and other offences against the person | 10 (1.1%) | 5 (2.1%) | 5 (0.7%) |
| Homicide and related offences | 5 (0.5%) | 0 (0.0%) | 5 (0.7%) |
| Sexual assault and related offences | 3 (0.3%) | 1 (0.4%) | 2 (0.3%) |

**Supplementary Figure 1.** Adjusted hazard ratios for violent reoffending by disruptive behavioural disorder and substance-induced psychosis status.





**Supplementary Figure 2.** Adjusted Hazard Ratios for (A) General Reoffending and (B) Violent Reoffending by Psychosis Status and Index Offense Type.

**
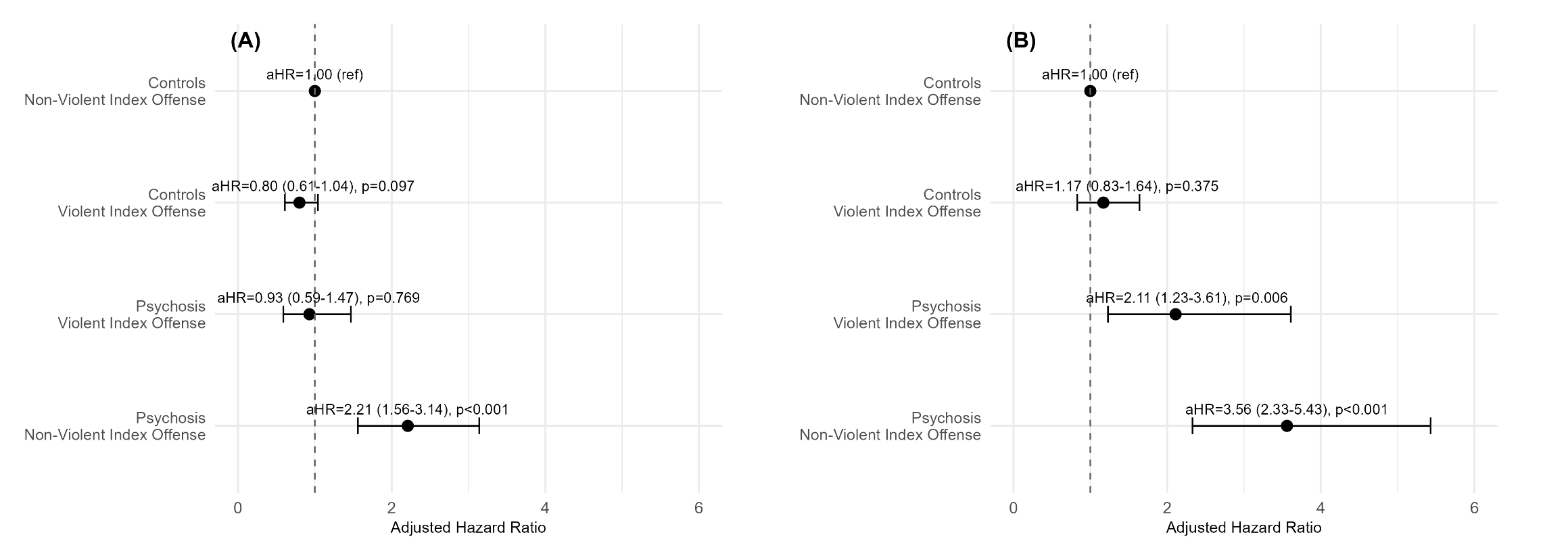
**
